# The Landscape of Regulatory Noncoding RNAs in Ewing’s Sarcoma

**DOI:** 10.3390/biomedicines9080933

**Published:** 2021-07-31

**Authors:** Connor Barrett, Anuj Budhiraja, Vijay Parashar, Mona Batish

**Affiliations:** Department of Medical and Molecular Sciences, University of Delaware, Newark, DE 19716, USA; cbarrett@udel.edu (C.B.); anujbmtl24@gmail.com (A.B.); parashar@udel.edu (V.P.)

**Keywords:** Ewing’s sarcoma, noncoding RNAs, microRNAs, long noncoding RNAs, regulatory RNAs, tumor progression, epigenetics, biomarkers, therapeutic targets

## Abstract

Ewing’s sarcoma (ES) is a pediatric sarcoma caused by a chromosomal translocation. Unlike in most cancers, the genomes of ES patients are very stable. The translocation product of the EWS-FLI1 fusion is most often the predominant genetic driver of oncogenesis, and it is pertinent to explore the role of epigenetic alterations in the onset and progression of ES. Several types of noncoding RNAs, primarily microRNAs and long noncoding RNAs, are key epigenetic regulators that have been shown to play critical roles in various cancers. The functions of these epigenetic regulators are just beginning to be appreciated in ES. Here, we performed a comprehensive literature review to identify these noncoding RNAs. We identified clinically relevant tumor suppressor microRNAs, tumor promoter microRNAs and long noncoding RNAs. We then explored the known interplay between different classes of noncoding RNAs and described the currently unmet need for expanding the noncoding RNA repertoire of ES. We concluded the review with a discussion of epigenetic regulation of ES via regulatory noncoding RNAs. These noncoding RNAs provide new avenues of exploration to develop better therapeutics and identify novel biomarkers.

## 1. Introduction

Ewing’s sarcoma (ES) primarily results in tumors of the bone or surrounding tissue and belongs to a larger group deemed Ewing’s sarcoma family tumors, which also includes peripheral primitive neuroectodermal tumors that affect mostly soft tissues [1,2]. ES accounts for roughly 10% to 15% of bone tumors and is the second most common malignant bone cancer in children [3]. Most ES cases occur between the ages of 15 and 20, with a slightly higher incidence in males [4]. Intermittent pain is a common symptom that tends to be first overlooked given the commonality of concurrent histories of trauma in children and adolescents, which may complicate and delay the diagnostic process [5]. Patient survival rates in localized cases are around 70% after five years but fall sharply to 30% after ten years and just 25% after five years if metastasis is present at diagnosis [6].

In 90% of cases, the onset of tumorigenesis is the fusion of the Ewing sarcoma break point region 1 (*EWSR1*) and Friend leukemia intergenic (*FLI1*) genes following a reciprocal translocation event [6]. *EWSR1* codes for an RNA-binding protein and is originally found on chromosome 22, while the *FLI1* proto-oncogene codes for a transcription factor and is originally located on chromosome 11. Translation of the fused genes results in a single chimeric product called EWS-FLI1, which contains the N-terminal transcription-activating domain of EWSR1 and the C-terminal DNA-binding domain of FLI1 [7]. EWS-FLI1 alters the expression of many genes involved in metabolic pathways and cell development, and it is the main oncogenic driver for ES [6,8]. Exploring mechanisms of action of ES remains an active field [9]. Significant research initiatives have pursued the identification of pathways in which EWS-FLI1 is involved in and the role of non-genetic/epigenetic contributors to these events [10,11,12]. Characterization of these epigenetic regulators, including noncoding RNAs, presents a promising new direction for future endeavors [13].

**Figure 1:**
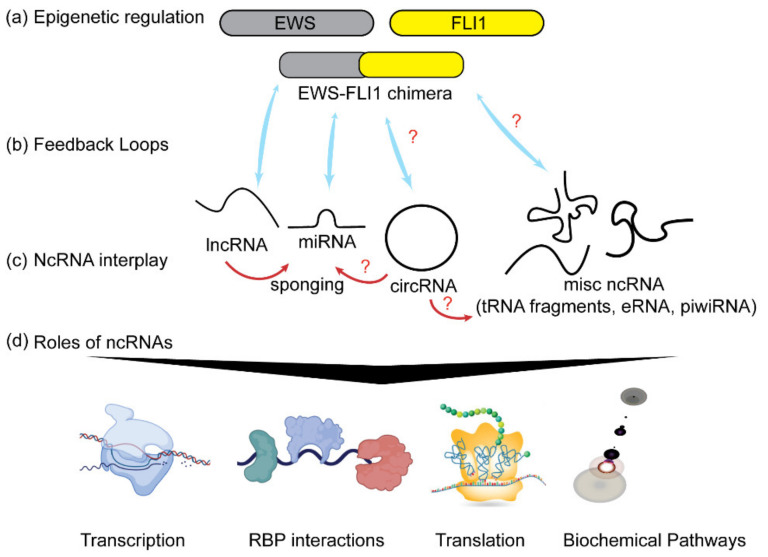
Noncoding RNAs and ES. (**a**) A chimeric fusion product of *EWS* and *FLI1* genes, EWS-FLI1, exerts epigenetic control by regulating the expression of different classes of ncRNAs (shown as blue arrows). (**b**) Some ncRNAs also alter the expression of EWSFLI1 as a means of feedback regulation of their own expression levels. (**c**) ncRNAs have an extensive interplay between each other (shown as red arrows). (**d**) ncRNAs play roles in regulating transcription, RNA export and transport. They interact with different RNA binding proteins (RBPs), translation and modulating signaling pathways. Red question marks indicate unknown and yet to be explored connections.

In this review, we aim to explore the role of regulatory RNAs in ES. A more thorough understanding of epigenetics could begin to explain why population-level differences (e.g., between European and African populations) exist and account for varied effects on the clinical level of treatment. Our comprehensive review of noncoding RNAs (ncRNAs) in ES covers primarily microRNAs (miRNAs) and long noncoding RNAs (lncRNAs); because other noncoding RNAs are not yet characterized for their role in ES. We based the structure of our literature search on the PRISMA 2020 statement flowchart design for systematic reviews (Figure S1). Though intended for systematic reviews, this flowchart was helpful in organizing search results. The numbers of included and excluded publications for each step are listed. We also include a report of initial keyword search results from PubMed in Table S1. Notably, other types of ncRNA like circular RNAs and piwiRNAs have been studied in the closely related osteosarcoma [14,15], but we were unable to locate any relevant publications linking them to ES; tRNA fragments and eRNAs have also been discovered in cancers other than ES [16,17,18]. Here, we present current understanding on the tumor suppressor roles of miRNAs, the oncogenic activity of both miRNAs and lncRNAs and the interplay between these two classes of ncRNAs in ES. The schematic of current state of knowledge and missing gaps is summarized in Figure 1.

## 2. MicroRNAs

### 2.1. Biogenesis and Function of MicroRNAs

MicroRNAs (miRNAs) are a specific type of noncoding RNA (ncRNA) averaging 22 nucleotides in length that play key functions in the epigenetic regulation of a variety of human processes and several cancers [19]. A number of pathways have been identified for the biogenesis of miRNAs. Briefly, there are four main steps in the biogenesis of miRNAs in the most well established canonical pathway [20]. In the first step, RNA polymerase II synthesizes long stem-loop structures called primary miRNAs (pri-miRNA) that have stable 7-methylguanosine caps and poly-(A) tails. In the second step, a microprocessor complex consisting of Drosha and associated factors like DiGeorge critical region 8 (DGCR8) interacts with the stem loop regions of the pri-miRNA and cleaves it into ~70 nucleotide long single hairpins called precursor miRNAs (pre-miRNAs). In third step, the pre-miRNAs are exported to the cytoplasm via exportin 5 (exp5), and the combination of Dicer and trans-activation-response RNA binding protein (TRBP) cleaves them further into ~21 nucleotide dsRNA duplexes. In the fourth step, an RNA-induced silencing complex (RISC) including an argonaute protein (AGO), results in one strand of miRNA being discarded, while the other remains with AGO as the mature functional miRNA product [21].

Usually miRNAs bind to the 3′ untranslated region (UTR) of target mRNA sequences, which results either in the degradation of target mRNA, or in the inhibition of translation of the target mRNA, thereby reducing gene expression [22]. MiRNAs act in a variety of biological pathways required for normal function including nervous system development, stem cell differentiation, angiogenesis and reproduction [23,24,25,26]. Previous reviews have indicated the ubiquity of miRNAs in a variety of sarcomas ranging from osteosarcoma, rhabdomyosarcoma, liposarcoma and many more. The dysregulation of cell cycle pathways, dysregulation of apoptosis, dysregulation of stemness and increased proliferation/migration/invasion tend to be the regulatory mechanisms pertinent to oncogenic miRNAs in sarcomas [27,28,29]. As with other cancers, certain miRNAs in ES are downregulated if they normally play a tumor suppressive role, while others are upregulated if they have oncogenic functions.

### 2.2. Tumor Suppressor miRNAs

The microRNAs whose normal expression in the cell is required to maintain the physiological states of the cell in terms of regulated cell growth and apoptosis are called tumor suppressor miRNAs. Some of these miRNAs are also involved in regulating the expression of oncoproteins. Here we describe a few of such tumor suppressor miRNAs reported to play tumor suppressive roles in ES. Ultimately, a plethora of studies showed several key examples of miRNAs that play protective roles, and downregulation of their expression often leads to progression of ES at the cellular level. 

#### 2.2.1. Let-7a and Associated miRNAs

A group of three miRNAs—let-7a-2-3p, 16-2-3p, and 29b-1-5p—is involved in an oncogenic loop with c-Myc and cyclin-D2 (CCND2) [30]. This is consistent with the established role of cell cycle regulation in preventing tumorigenesis [31]. Both c-Myc and CCND2 were found to be overexpressed in ES-derived cell lines, and it was subsequently found that c-Myc negatively regulates all three miRNAs, while CCND2 is the downstream target. This oncogenic loop illustrates how miRNA connects regulators and targets. In ES, the c-Myc pathway along with the spleen tyrosine kinase (SYK) pathway can promote the transcription of the long noncoding RNA metastasis associated lung adenocarcinoma transcript 1 (MALAT1) [32,33]. MALAT1 has a variety of oncogenic functions in many cancers including breast cancer to renal cell carcinoma and often correlates with decreased survival rates [33]. In non-cancerous cells, let-7a-2-3p, 16-2-3p, and 29b-1-5p also act to suppress oncogenesis by retarding the cell cycle at G0/G1 [30]. The robustness of this miRNA group’s tumor suppressor model was further tested by an ex vivo quantification of tumor size in mouse xenograft models, which further confirmed the tumor suppressor effects of these miRNAs [30]. Let-7a itself also acts in an additional regulatory axis with signal transducer and activator of transcription 3 (STAT3) and phospho-NF-KB p65 (p-P65) to inhibit carcinogenesis, including control over macrophage infiltration [30]. STAT3 was shown to be a target of let-7a, and STAT3 inhibition resulted in reduced ES cell proliferation [34]. The activated p-P65 component of the NF-κB pathway was directly related to STAT3; thus, with let-7a inhibition in ES cells, carcinogenic activities of both STAT3 and p-P65 were upregulated [34]. Supporting the oncogenic effects of STAT3 in ES, it has been shown that STAT3 affects the surrounding tumor microenvironment through chemokine regulation and generally reduces tumor viability in ES cell lines when inhibited, as investigated by cell viability growth assays [35]. Like MALAT1, STAT3 dysregulation is not unique to ES due to its many roles in proliferation, migration, angiogenesis and microenvironment conditions in a variety cancers, and it is a popular target for a multitude of pharmaceutical inhibitors [36].

#### 2.2.2. miR-145 and miR-143

Though several miRNAs are closely related to EWS-FLI1 regulation (let-7g, miR-22, miR-30a-5p) [37,38,39], miR-145 was the sole miRNA identified to directly bind to the 3′-UTR of FLI1 [40]. Consequently, it was seen that the lower levels of miR-145 in ES cells were associated with greater levels of EWS-FLI1 expression. Other targets of miR-145, particularly the ones related to stemness including octamer-binding transcription factor 4 (Oct4), SRY+Box transcription factor 2 (SOX2), Kruppel Like Factor 4 (KLF4) and Myc were also found at higher levels in ES. This indicated that EWS-FLI1 controls both the degree of its autoregulation and these associated oncogenic pathways. Specifically, Oct4 interacts with the Ewing’s sarcoma protein EWS directly enhancing differentiation [41], whereas SOX2 enhances proliferation through cell cycle and apoptosis dysregulation [42]. KLF4 remains less studied specifically in ES, but its expression has been observed at higher levels in other cancers like breast and gastrointestinal cancer [43,44]. In addition to miR-145, the closely related miR-143 was found to have tumor suppressing roles in ES, targeting the stemness factor Nanog, in addition to those previously listed [45]. Although the exact mechanisms of downregulation of miR143 and miR145 in ES are not well understood, it is speculated that EWSFLI1 is regulating miRNA processing pathways. It was found that expression of miRNA processing enzyme TARBP2 is reduced in ES [45]. Further, treatment with methylation-affecting drugs including 5-Azacytidine (5-AzaC) and 3-deazaneplanocin A (DZNep) returned TARBP2 levels to normal, which simultaneously led to restoration of proper miR maturation functions for miR-143 and miR-145. It has been previously demonstrated that 5-AzaC inhibits DNA methylation and that DZNep inhibits histone methylation [46,47]. Overall, the interaction between these inhibitors and miRNA presents a new and intriguing intersection between clinical level treatments directly affecting the methylation and epigenetics of ES.

#### 2.2.3. miR-124

Another critical tumor suppressor miRNA in ES is miR-124. Its downregulation has been shown to cause increased cell growth and motility, increased mesenchymal differentiation and increased invasion [48]. Its targets include snail family transcriptional repressor 2 (SNAI2, also known as SLUG), a suppressor of epithelial markers E-cadherin and ꞵ-catenin, as well as G1 to S cell cycle regulator CCND2. SLUG is related to mesenchymal markers and increased oncogenesis in other cancers such as prostate and breast cancer [49,50]. It was also previously discovered that SLUG downregulates E-Cadherin expression and is partly responsible for the cellular transition from epithelium to mesenchymal [51]. More specifically, downregulation of E-Cadherin was observed in ES cell lines and shown to be inversely related to SLUG levels [48]. In accordance with the established cell cycle regulatory role of CCND2, miR-124 upregulation consequently resulted in increased G1-S cell cycle arrest [48]. In vivo studies further confirmed the tumor suppressor role of miR-124 by indicating higher rates of ES metastasis [48]. Interestingly, the epigenetic control by which miRNA suppression is carried out appears similar to that of miR-143 and miR-145. Namely, the drug 5-Aza-CdR was able to recover the expression of miR-124, though it should be noted that direct functional characterization of the miRNA maturation pathway and the exact mechanisms of methylation regulation have not yet been elucidated for miR-124 [48]. Table 1 lists these and other known tumor suppressive miRNAs reported in ES.

### 2.3. Oncogenic miRNA

This class of miRNAs is usually not expressed or expressed at very low level in normal cells. However, the onset of tumorigenic factors leads to an upregulation of these oncogenic miRNAs (oncomiRs). OncomiRs often function by suppressing the expression of tumor suppressor proteins and cell cycle regulators. OncomiRs also generally promote survival of the tumor by enhancing resistance to therapy by downregulating targets involved in therapeutic responses [70]. A handful of miRNAs have been reported to play oncogenic roles in ES, which presents a notable contrast to the larger number of tumor suppressive miRNAs. We discuss a few of these key oncomiRs and provide a list of the relevant oncogenic miRNAs reported in ES.

#### 2.3.1. miR-181c-5p and miR-193a-5p

Investigation into the oncogenic functions of both miR-181c and miR-193a-5p have revealed that both have varied but interconnected roles in anti-apoptotic mechanisms of ES. Cisplatin treatment appeared to increase the levels of cleaved poly (ADP-ribose) polymerase (PARP), which can be taken as a measure of apoptosis [71,72]. Both of these miRNAs were linked with lower levels of PARP cleavage, suggesting reduced apoptosis, even though their intermediate targets were different; Fas cell surface death receptor (FAS) for miR-181c and trans activating p73 beta (TAp73β) for miR-193a-5p. FAS requires binding of its ligand for apoptotic activation, but dysfunctional FAS ligand has been previously observed in ES [73,74]. With the addition of this more recent miRNA discovery, it appears multiple mechanisms work to inhibit apoptosis via FAS regulation. Similarly, TAp73 is a known tumor suppressor in other cancers and its downregulation has been linked to increased activity of the NF-κB pathway in breast cancer as well as increased stem factor expression of Oct4, nanog and SOX2 in carcinoma-like stem cells [75]. Ultimately, both of these miRNAs lead to dysfunction of caspase pathway activity thereby preventing apoptosis and facilitating increased tumor growth [71,73].

#### 2.3.2. miR-210-3p

MiR-210 was found to directly target and reduce caspase 8 associated protein 2 (CASP8AP2) levels and thus has implications in the FAS pathway as well [76]. In addition to these shared pathways with miR-181c and miR-193a-5p, miR-210 had an interesting role in exosomal miRNA delivery in ES. It was found that a hypoxic tumor microenvironment (TME) increased cancerous sphere formation, and that miR-210 expression was enriched in the hypoxic spheres. This appeared to have a positive feedback mechanism in that miR-210 further promoted the production of spheres [76]. A hypoxic TME promotes the expression of hypoxia-inducible-factor 1 and 2 (HIF1 and HIF2) [77]. It has been suggested that hypoxia generally promotes more aggressive cancer by encouraging metastasis and vascularization through HIF1 and HIF2 but that p53 antagonistic effects adds complexity to the model [78]. Accordingly, HIF1 and HIF2 were found to be elevated by hypoxia in both osteosarcoma (OS) and ES. While apoptosis was enhanced in both cancer cell lines, migration was only enhanced in OS [77]. Clearly, the role of both exosomal miRNA and the hypoxic TME in ES is still a developing field, but such results with miR-210 indicate that there are certainly novel functions to explore in this direction. Table 2 lists these and other oncogenic miRNAs reported in ES.

## 3. Long Noncoding RNAs

### 3.1. Biogenesis and Function of lncRNA

Long noncoding RNAs (lncRNAs) tend to be at least 200 nucleotides long and are one of the many RNA subclasses without a protein-coding potential. They have a variety of functions including chromatin remodeling, translational regulation and gene silencing, among other critical functions which affect a multitude of cellular pathways and protein expression [85]. The range of downstream effects induced by the altered expression of lncRNAs has implications in neuronal disorders such as Huntington’s disease and lateral amyotrophic sclerosis, autoimmune conditions, and cancers [86,87]. The biogenesis of lncRNAs is well understood: lncRNAs are transcribed by RNA Polymerase II from poorly conserved regions of the genome, share some of the structural features of mRNAs and may undergo alternative splicing [88]. The characterization of lncRNAs in ES is still in its infancy. There are only a few research articles that identified expression of lncRNAs in ES, and an even smaller fraction of lncRNAs are characterized for their function in ES [85]. Below we discuss the characterized lncRNAs followed by a table of key reported lncRNAs in ES that primarily participate in post transcriptional regulatory mechanisms [85].

#### 3.1.1. lncRNA EWSAT1

Long noncoding RNA-227 or lncRNA Ewing Sarcoma-associated transcript 1 (EWSAT1) was the first lncRNA discovered in ES as a result of RNA sequencing analysis. EWSAT1 was found to be upregulated in primary pediatric human mesenchymal progenitor cells which are the likely origin cells for ES development. Evidence indicates that EWSAT1 is a downstream target of the EWS-FLI1 product as EWSAT1 levels were decreased after EWS-FLI-1 knockdown, whereas they are upregulated in ES cell lines. Subsequent EWSAT1 experiments have supported the hypothesis that EWSAT1 promotes proliferation in-vitro, but the mechanisms by which it regulates gene expression are thought to be diverse due to its localization in both the cytoplasm and the nucleus. EWSAT1, which plays a role in the regulation of 404 reported genes, is primarily a mediator of gene repression and has significant overlap in its target genes with the EWS-FLI1 product [89]. Though the identification of this lncRNA’s mechanisms in ES cells is a gap in the existing literature, EWSAT1 has since been found to be upregulated in osteosarcoma, cervical cancer, and colorectal cancer among others, in which its suggested mechanism is as a competing endogenous RNA. For example, in colorectal cancer, EWSAT1 sponges miR-326 to upregulate FBXL20 and promote proliferation, migration and invasion [90]. Similarly in osteosarcoma, EWSAT1 sponges miR-24-3p and disrupts the miR-24-3p/ROCK1 axis to promote metastasis [91].

#### 3.1.2. lncRNA HULC

Long noncoding RNA, Highly Upregulated in Liver Cancer (HULC), is a lncRNA which is highly expressed in ES cell lines. Knockdown of HULC significantly hindered ES cell proliferation and clonogenicity; however, apoptosis was not induced. Mechanistically, HULC shares its cytoplasmic localization and suggested function as a competing endogenous RNA with SOX2OT. Several miRNA binding sites within HULC’s sequence have been identified, but in-silico analysis has identified miR-186-5p to have the highest binding score [92]. The tumor suppressive miR-186-5p can bind to twist basic helix-loop-helix transcription factor 1 (TWIST1) transcripts and inhibit their translation as the two RNAs are negatively correlated [92]. TWIST1 plays a role in ES metastasis, as its suppression reduces metastasis but does not affect the development of the original tumor [93]. HULC’s sponging effect on miR-186-5p was established in the evaluation of the small molecule YK-4-279, which reduces the transcriptional activity of EWS-FLI1 [94,95]. Treatment of ES cell lines with YK-4-279 downregulated HULC, decreasing its sponging effect on miR-186-5p thereby increasing TWIST1 transcript binding with miR-186-5p and reducing TWIST1 protein levels [92].

#### 3.1.3. lncRNA SOX2OT

The lncRNA SOX2OT is a recognized oncogene in many human carcinomas such as colorectal and gastric cancer, and RT-PCR results indicated that SOX2OT transcript expression is also elevated in ES cell lines [96]. SOX2OT is suggested to promote malignant behaviors such as proliferation, cell invasion and survival as these mechanisms were suppressed upon in-vitro transfection of ES cells with si-SOX2OT. SOX2OT is localized in the cytoplasm where it has interplay with miR-363, a miRNA which has suggested anti-carcinogenic effects in other cancers such as gliomas [96]. Another target regulated by SOX2OT is the transcription factor FOXP4 (forkhead box p4) which is involved in several oncogenic axes [97]. Though SOX2OT does not bind directly to FOXP4 mRNA, it modulates the effect of miR-363 on FOXP4 protein expression. Specifically, SOX2OT functions as a competing endogenous lncRNA, sponging miR-363 as supported by luciferase assay results which established the negative correlation between the two noncoding RNAs. Significant upregulation of SOX2OT, as is found clinically in ES samples, decreases miR-363 levels and therefore increases FOXP4 protein levels, inducing many downstream malignancy-promoting pathways [98]. A list of these and other lncRNAs studied in ES is provided in Table 3.

## 4. Future Perspectives

Consistent with the widespread use of bioinformatic data in the modern scientific enterprise, many papers reviewed had extensive supplementary information regarding more miRNA than were reported here. From differential expression profiles, a subset is investigated further using RNA-seq, and an even smaller subset is chosen as the primary candidates for research papers. Thus, there are a multitude of miRNAs and lncRNAs that have yet to be fully validated for their function in ES.

### 4.1. Unexplored Connections

**Figure 2:**
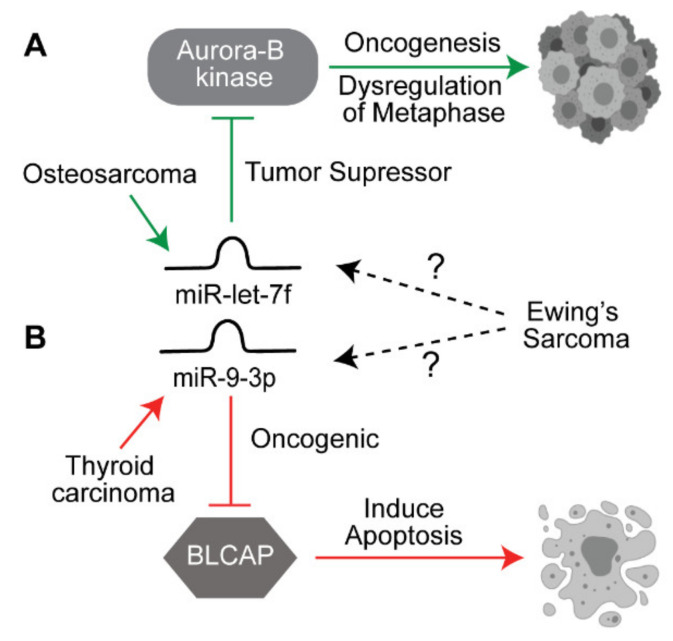
Potential link between miRNAs identified in other cancers. (**A**) miR-let7f acts as a tumor suppressor miRNA by downregulating the oncogene Aurora B kinase in osteosarcoma. (**B**) miR-9-3p acts as an oncogenic miRNA by suppressing BLCAP which is required for inducing apoptosis in thyroid cancer. Both of these target genes are similarly affected in ES, but the role of these miRNAs is not yet elucidated in ES. The solid arrows indicate known pathways, the dotted arrows indicate yet to be explored pathways.

There currently exist many unexplored connections involving uncharacterized ncRNA and ES. Examples include miRNA dysregulated pathways shared between cancers that currently have missing links to ES (Figure 2). For example, the EWS protein is responsible for deregulating metaphase activity in mitosis via an up regulatory interaction with the Aurora-B kinase, which is a suspected oncogenic mechanism [100,101]. The tumor suppressor miRNA let-7f was found to target Aurora-B to reduce proliferation, invasion and migration but was only researched in OS [102]. Given that OS is related to ES, it seems plausible that let-7f may be downregulated in ES given a shared mechanism between the two; however, this link has not been reported in the current research. Another example is miR-9-3p, which has been reported to target apoptotic factor Bladder cancer-associated protein (BLCAP) and therefore act as an oncogenic anti-apoptotic driver in thyroid carcinoma [103]. In ES, BLCAP has been shown to be an inducer of apoptosis [104]. Thus, upregulation of miR-9-3p is another hypothetical connection yet to be studied in ES. It is therefore worth to carefully look at ncRNAs identified in other tumors that have implications in shared oncogenic mechanisms but no conclusive links in ES.

### 4.2. Biomarkers

A low expression level of miR-34a has been shown to be correlated with worse ES survival rates [57]. Consistent with this observation, it was found that ES tended to recur in patients with low miR-34a, while higher expression was linked with higher survival rates, reduced relapse and reduced metastasis [58]. Independently, the presence of metastasis at diagnosis was correlated with lower survival rates [105]. Future studies may elucidate if a miRNA differential profile, such as that of miR-34a, precedes traditional clinical diagnoses, which would aid in the delivery of earlier treatment and better outcomes. Further, as the characterization of exosomal RNA cargo (e.g., exosomal miRNAs) is being expanded to various tumors, it is only a matter of time before more regulatory ncRNAs could be identified in the exosomes to serve as non-invasive diagnostic biomarkers to predict the onset, progression, and recurrence of ES.

### 4.3. Other Regulatory RNAs

As mentioned before, no direct studies were found exploring the role of other ncRNAs; however, there is an upcoming interest to explore the vast repertoire of other emerging regulatory RNAs. One of the recently appreciated classes is circular RNAs. CircRNAs are produced by back-splicing events and as their name suggests these are closed ended covalent circles [106]. CircRNAs are resistant to exonucleases and have emerged as clinically relevant regulatory RNAs in several cancers including osteosarcoma and other pediatric cancers [107]. The role of circRNAs has not been reported in ES directly yet. Though there is one reported circRNA called circEF, an oncogenic fusion associated circRNA, its mechanism of action has not been explored [108]. The major function of circRNAs is to sponge miRNAs and thus regulate mRNA expression [109]. The circRNAs act as the regulator of miRNA-mRNA axes as done by some of the characterized lncRNAs. Furthermore, circRNAs are highly enriched in exosomes making them excellent biomarkers [110]. It is only a matter of time when circRNAs and the other uncharacterized ncRNAs will be explored in ES.

## 5. Conclusions

Noncoding RNAs can act as network modulators, the targets of unidentified lncRNA/circRNA sponge axes or players in yet unidentified cellular roles. The recent appreciation of ncRNA packaging and release via extracellular vesicles has further made these regulatory RNAs key players in maintaining the tumor microenvironment and modulating cell to cell communication. The field of regulatory RNAs is growing exponentially for all tumors, including pediatric sarcomas. Just last month, a new study by Chen et al. used machine learning and training models on existing RNA sequencing data sets from ES patients to identify a set of seven lncRNAs as prognostic risk marker for ES. The increased expression of these seven lncRNAs was statistically correlated with poor overall survival [111]. These seven lncRNA, including SNHG17, WAC-AS1, LINC00623, SSBP3-AS1, and TDRG1, are yet to tested experimentally for their association and role in ES, although some have functional roles in other cancers [111]. This study opened a new research direction to explore the use of a set of ncRNAs as prognostic markers. This could potentially also include a set of different regulatory RNAs as a signature for the progress of the disease. In conclusion, this review aimed to provide an assessment of the current landscape of ncRNA, to emphasize the unmet need for research in this field, to explore novel candidates and to characterize the function of existing targets.

## Figures and Tables

**Table 1:** MicroRNA in Ewing’s Sarcoma with Tumor Suppressor Functions.

Name	Functions	Pathways	Targets	Citation
Let-7a-2-3p, miR-16-2-3p, miR-29b-1-5p	G0-G1 arrest	c-Myc	CCND2	[30]
Let-7g	Let-7g processing	EWS-FLI1/Drosha	Dicer, CCND1	[37]
miR-22	Anchorage dependent growth, histone methylation	EWS-FLI1	KDM3A	[38]
miR-30a-5p	Proliferation, Invasion	EWS-FLI1	CD99	[39]
miR-143/145	Stemness	EWS-FLI1	FLI1 Oct4, Sox2, Klf4, Myc, Nanog	[40,45]
miR-124	Mesenchymal-epithelial markers G1-S arrest	CCND2	CCND2SLUG	[48]
miR-15a	Growth inhibition	? ^1^	CCND1, Bcl-2	[52]
miR-21-3p	Proliferation, metastasis	? ^1^	ALCAM/CD166	[53]
miR-27a, miR-100	General oncogenesis	IGF	IGF-1	[54]
miR-125b	General oncogenesis	IGF RSK1	IGF-1	[54]
miR-30d	S-phase arrest	MEK/ERK and PI3K/Akt	MMP-2 MMP-9	[55]
miR-31	Proliferation Invasiveness	? ^1^	? ^1^	[56]
miR-34a	Proliferation, chemo-sensitization	P53	CCND1, Bcl-2	[57]
	Proliferation	? ^1^	CCND1	[58]
	Differentiation	Notch, NF-κB	Notch1, Delta	[59]
miR-107	Proliferation Tube formation Cell cycle arrest Apoptosis	HIF	HIF-1β	[60]
miR-124-3p	Cell cycle inhibition	DLX6-AS1/miR-124-3p/CDK4 axis	CDK4	[61]
miR-124-3p, 139-5p, 584-5p	Invasion, migration	? ^1^	ROCK1	[62]
miR-125b	Proliferation, migration, invasion	P13K/Akt	PIK3CD	[63]
miR-138-1-3p	Cell cycle repression Adhesion repression (anoikis)	FAK	FAK	[64]
miR-185	Bcl-2Bax	PI3K/AKT and Wnt/β-catenin	E2F6	[65]
miR-193b	Anchorage dependent growth	ErbB4	ErbB4	[66]
miR-199b-5p	Proliferation, invasion, cell cycle, apoptosis, G1-S arrest	? ^1^	CCNL1	[67]
miR-638	Tube formation Cell cycle arrest	VEGF	VEGFA	[68]
miR-708	DNA repair	EYA chemoresistance	EYA3	[69]

^1^ ? = Unknown role. Most studies of tumor suppressor miRNA in ES focused on cell line assays and experiments with the following exceptions being noted: investigations into miR-21-3p and miR-34a used primary tumor data sets [53,57]; miRNA expression can decrease with disease progression (e.g., metastasis) for miR-34a, miR-139-5p and miR-584-5p [58,62]; miRNA expression at other times remain statistically equal across disease progression as in the case of miR-124-3p [62].

**Table 2:** MicroRNAs in Ewing’s Sarcoma with Oncogenic Functions.

Name	Functions	Pathways	Targets	Citation
miR-106a~363	Pro-growth	? ^1^	?	[52]
miR-193a-5p	Anti-apoptosis, cisplatin chemoresistance	Caspase 3/7, PARP	TAp73β	[71]
miR-181c-5p	Anti-apoptosis	Caspase 3/7/8, PARP	FAS	[73]
miR-210-3p	Exosomes in hypoxic TME, sphere formation, anti-apoptosis	FAS and TNF-α	CASP8AP2	[76]
miR-20b-5p	Cell cycle, Anti-apoptosis, pro-proliferation	TGF-ꞵ, MYC, Smad	TGFBR2	[79]
miR-34b	Pro-growth, migration, invasion	Notch	Notch1	[80]
miR-125b-1, miR-125b-2	Chemoresistance	p53/Bak	P53, Bak	[81]
miR-130b	Pro-proliferation, invasion, migration	CDC42/PAK1/JNK-AP1	ARHGAP1	[82]
miR-146b-5p	Pro-proliferation, invasion, migration	? ^1^	BTG2	[83]
miR-301a-3p	G0/G1 checkpoints, anti-apoptosis, malignancy	P13K/Akt	PTEN	[84]

^1^ ? = Unknown role. With the exception of miR-34b, which utilized tissue sample data, other studies investigated oncoMiRs in cell lines [80].

**Table 3:** LncRNA in Ewing’s Sarcoma.

Name	Functions	Pathways	Targets	Citation
MALAT1	Pro-growth Pro-proliferation, angiogenesis, migration	SYK/c-MYC/ MALAT1 Hippo	EZH2	[32]
lncRNA DLX6-AS1	Pro-proliferation, Pro-cell survival	DLX6-AS1/miR-124-3p/CDK4	MiR-124-3p	[61]
EWSAT1	Pro-proliferation	? ^1^	HNRNPK	[89]
HULC	Pro-growth Chemoresistance Pro-proliferation	HULC/ miR-186-5p/ TWIST1	miR-186-5p	[92]
lncSOX2OT	Metastasis, Pro-proliferation, invasion, migration	SOX2OT/miR-363/FOXP4	miR-363	[98]
pncCCND1_B	Pro-growth, Pro-proliferation	? ^1^	Sam68	[99]

^1^ ? = Unknown role.

## Data Availability

All the data is provided in the manuscript and the supplementary data.

## Supplementary Materials

The following are available online at https://www.mdpi.com/article/10.3390/biomedicines9080933/s1, Figure S1: Adapted PRISMA 2020 flow diagram for a review including searches (e.g., “Ewing’s sarcoma” + miRNA) of databases only; Table S1: PubMed Keyword Search Totals.
