# Supplementary material for: The Landscape of Regulatory Noncoding RNAs in Ewing’s Sarcoma
Source: Biomedicines. 2021 Jul 31;9(8):933. doi: 10.3390/biomedicines9080933 (PMC8391329; doi:10.3390/biomedicines9080933)
Supplement: Supplementary file 1 [file biomedicines-09-00933-s001.zip › biomedicines-1289785-supplementary.pdf]

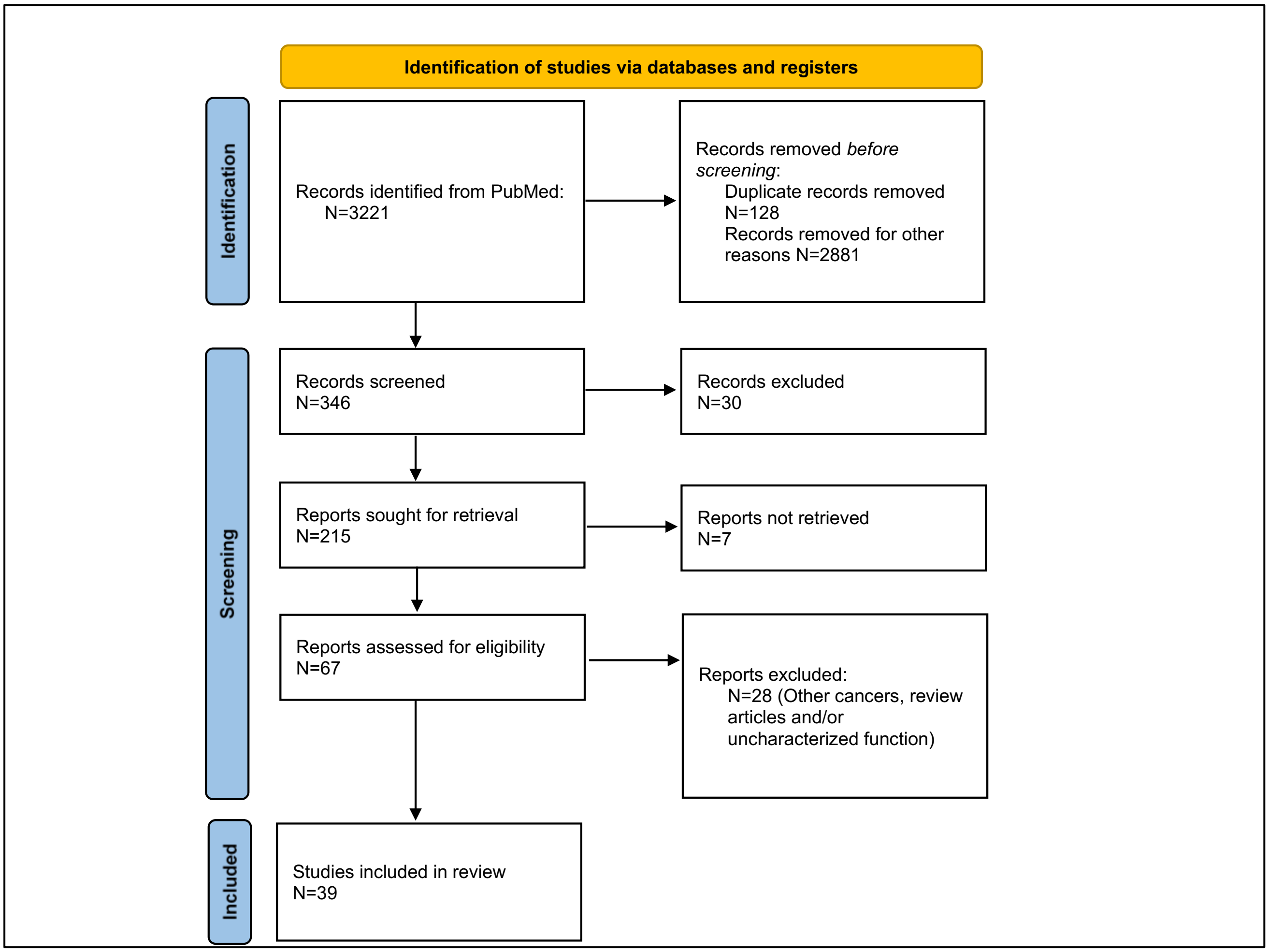


**Figure S1.** Adapted PRISMA 2020 flow diagram for a review including searches (e.g. "Ewing's sarcoma" + miRNA) of databases only. Numbers do not include articles relevant to background information, ES pathology or hypothetical connections.

**Table S1.** PubMed Keyword Search Totals

| Search Terms (2011-2021 Filter) | Results |
|---|---|
| ncRNA AND Ewing's sarcoma | 124 |
| miRNA AND Ewing's sarcoma | 84 |
| circRNA AND Ewing's sarcoma | 1 |
| tRNA fragments AND Ewing's sarcoma | 2 |
| siRNA AND Ewing's sarcoma | 79 |
| splicing AND Ewing's sarcoma | 33 |
| lncRNA AND Ewing's sarcoma | 17 |
| biomarkers AND Ewing's sarcoma | 500 |
| diagnosis AND Ewing's sarcoma | 2381 |
